# Primal-dual approach to optical tomography with discretized path integral with efficient formulations

**DOI:** 10.1117/1.JMI.4.3.033501

**Published:** 2017-07-19

**Authors:** Bingzhi Yuan, Toru Tamaki, Bisser Raytchev, Kazufumi Kaneda

**Affiliations:** Hiroshima University, Department of Information Engineering, Graduate School of Engineering, Higashi-Hiroshima, Japan

**Keywords:** optical tomography, multiple scattering, path integral, primal-dual interior point method

## Abstract

We propose an efficient optical tomography with discretized path integral. We first introduce the primal-dual approach to solve the inverse problem formulated as a constraint optimization problem. Next, we develop efficient formulations for computing Jacobian and Hessian of the cost function of the constraint nonlinear optimization problem. Numerical experiments show that the proposed formulation is faster (23.14±1.32  s) than the previous work with the log-barrier interior point method (91.17±1.48  s) for the Shepp–Logan phantom with a grid size of 24×24, while keeping the quality of the estimation results (root-mean-square error increasing by up to 12%).

## 1 Introduction

Optical tomography^1^^–^^8^ is a technique for reconstructing inside an object by illuminating it with a light probe and observing the light penetrating through the object. In contrast to x-ray computed tomography, which uses x-rays instead of light, safer tomographic methods are demanded and scattering optical tomography methods are recently attracting computer vision researchers’ attention.^9^^–^^15^

We investigate a recently proposed optical tomography with discretized path integral.^12^^,^^13^^,^^15^ Their method takes advantages of the path integral formulation and formulates the inverse problem of optical tomography as a constraint nonlinear least squared optimization problem. This method benefits from various optimization techniques, which is not the case for voting^11^^,^^14^ or genetic algorithms.^10^ They solved the constraint optimization problem by using the log-barrier (LB) interior point method^16^ with inner loops of Newton method^12^ and quasi-Newton method.^15^ They have shown that optical tomography with discretized path integral produces better estimation results compared to a standard diffuse optical tomography (DOT),^15^ however, its high computation cost is a problem for further development.

In this paper, we propose two contributions to tackle the problem. First, we introduce the primal-dual (PD) approach to solve the constraint optimization because it is known that the PD interior point method is more efficient than the LB method.^16^ Second, we propose new formulations of equations. The main bottle-neck of the previous approaches^12^^,^^13^^,^^15^ is Jacobian and Hessian which are computationally demanding as the number of paths increases. Our new formulations are equivalent to the previous ones, but much more efficient. Numerical simulations show that the proposed approach accelerates the estimation by a factor of 100. (Conference versions of this paper were presented.^17^^,^^18^ This paper extends those versions with the extended description of the PD approach and the efficient formulations, and additional experiments with optimized codes with comparisons.)

There exists a number of acceleration methods for tomographic reconstruction; such as dimension reduction,^19^ power acceleration,^20^ and, most importantly, graphic processing units (GPUs).^21^ GPUs have been used for accelerating a forward problem^22^ and forward and backward projections.^23^^–^^25^ In addition, thanks to the recent progress of general-purpose computing on GPUs, GPUs have become popular for speeding up iterative computations for solving compressive sensing formulations^26^ and large linear systems.^27^^,^^28^ We have not implemented the proposed approach with any GPUs in this paper, however, the use of GPUs would be beneficial for the proposed formulations because computing a Jacobian matrix can be further accelerated.^29^

In the following, we briefly summarize the constraint problem to be solved in Sec. 2. Then in Sec. 3, we develop the PD method to solve the problem by taking into account the structure of the problem. In Sec. 4, we derive new efficient formulations to compute Jacobian and Hessian, with comparisons to and discussions of the previous formulations. We show simulation results in Sec. 5 to show the improvement of the proposed method in terms of computation cost.

## 2 Preliminary

In this section, we briefly review the formulation of optical tomography with a discretized path integral. Details can be found in Ref. 15.

**Fig. 1:**
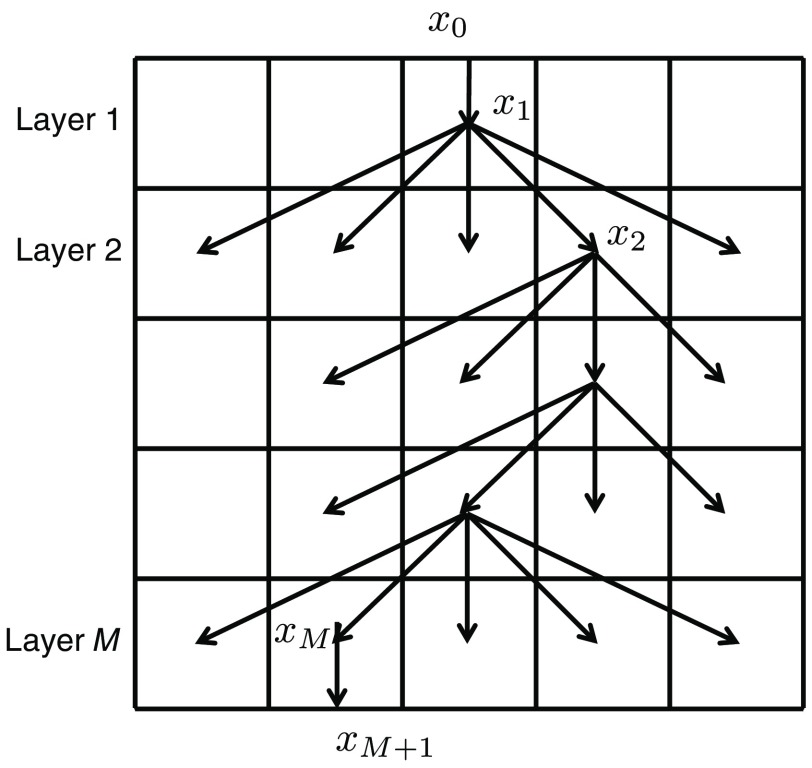
2-D layered model of scattering.^15^ This example of a 5×5 grid shows a path consisting of vertices $x_{1},\ldots ,x_{M}$ located at the centers of voxels in a grid with M parallel layers. $x_{0}$ is a light source located on the top surface, and $x_{M+1}$ is a detector at the bottom.

We are interested in estimating the extinction coefficients $\sigma_{t}$ of each voxel in a two-dimensional (2-D) medium as shown in Fig. 1. Extinction coefficients represent how much light attenuates at each point. We follow the 2-D layer model,^15^ that is, we assume the following layer scattering with the following properties. Suppose that the 2-D medium has a layered structure and is discretized into voxels of an M×N grid; it has M layers of N voxels. Therefore, the problem is to estimate extinction coefficients of each voxel in the grid.

**Fig. 2:**
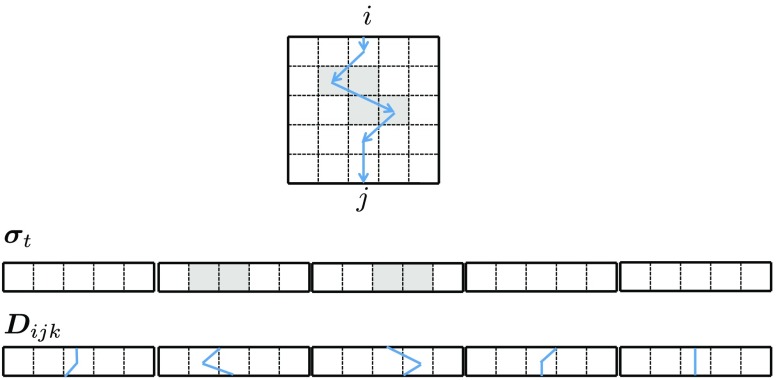
An example of a path in a 5×5 grid. The path $D_{ijk}$ is one of paths that connect locations i and j. The grid is serialized to vector $\sigma_{t}$, and also to vector $D_{ijk}$ separately, but in a consistent order. These vectors are used to represent the exponential attenuation of light along the path by inner product followed by the exponential function as $\exp(-\sigma_{t}^{T}D_{ijk})$ in Eq. (3).

With this layered medium, we use an observation model of the light transport between a light source and a detector: emitting light to each of the voxels at the top layer and capturing light from each voxel from the bottom layer (see Fig. 2). More specifically, the light source point $x_{0}$ is located on the boundary of the top surface of the voxels in the top layer. The detector point $x_{M+1}$ is located on the boundary of the bottom surface of the voxels in the bottom layer. Then, forward scattering happens layer by layer; light is scattered at the center of a voxel in a layer and goes to the center of a voxel in the next (below) layer. By connecting the centers $x_{1},\ldots ,x_{M}$ of voxels of each layer, we have a path $x_{0},x_{1},\ldots ,x_{M},x_{M+1}$ of light scattering connecting the light source and the detector. Let i and j be voxel indices of the light source and detector locations, respectively. By restricting the light paths only to those connecting i and j, the observed light $I_{ij}$ by the detector is the sum of contributions of all paths connecting i and j. This is written as follows:

$$I_{ij}=I_{0}\overset{N_{ij}}{\underset{k=1}{\sum }}H_{ijk}e^{-\sigma_{t}^{T}D_{ijk}},$$ (1)

where $\sigma_{t}\in R^{NM}$ is a vector to be estimated, and each element is the extinction coefficient of a voxel, as shown Fig. 2. Vector $D_{k}\in R^{NM}$ represents a complete path k connecting i and j, and each element is the length of the part of the path segment passing through the corresponding voxel. Factor $H_{ijk}$ encodes scattering coefficients and the phase function, $I_{0}$ is the intensity of the light source, and $N_{ij}$ is the number of paths connecting i and j. Parameters $H_{ijk}$, $I_{0}$, $N_{ij}$, $D_{k}$ are given and fixed. The problem is to estimate $\sigma_{t}$ based on observations $I_{ij}$.

By changing positions of the light source i and the detector j, we obtain a set of observations $\left\{I_{ij}\right\}$, resulting in the following nonlinear least squares problem under box constraints for extinction coefficients to be positive

$$\underset{\sigma_{t}}{\min}\;f\;\mathrm{s.t.}\;\sigma_{tl}\leq \sigma_{t}\leq \sigma_{tu},$$ (2)

where f is the cost function

$$f=\overset{N}{\underset{i=1}{\sum }}\overset{N}{\underset{j=1}{\sum }}{\left|I_{ij}-I_{0}\overset{N_{ij}}{\underset{k=1}{\sum }}H_{ijk}e^{-\sigma_{t}^{T}D_{ijk}}\right|}^{2},$$ (3)

and $\sigma_{tl}$ and $\sigma_{tu}$ are lower and upper bounds, respectively. The box constraints are due to the nature of the extinction coefficient being positive (i.e., $\sigma_{tl}>0$) and the numerical stability of excluding unrealistic large values.

## 3 Primal-Dual Interior Point Method of the Inverse Problem

Here, we develop a PD interior point method to solve the inverse problem [Eq. (2)]. It is an inequality constraint optimization with box constraints, which is straightforward in applying a standard PD method.^16^ However, we can use the structure of the box constraints, hence, we derive an efficient algorithm by using the problem structure.

### 3.1 Primal-Dual Method

We first rewrite the inequality constraint problem to an equivalent problem with equality constraints with slack variables $s={\left(s_{1},\ldots ,s_{2NM}\right)}^{T}$ as follows:

$$\underset{\sigma_{t}}{\min}\;f\;\mathrm{s.t.}\;c-s=0,\;0\leq s,$$ (4)

where c is a vector of the box constraints

$$c=(\begin{array}{c} c_{1}(\sigma_{t})\\ \vdots \\ c_{2NM}(\sigma_{t}) \end{array})=(\begin{array}{c} \sigma_{t}-\sigma_{tl}1\\ \sigma_{tu}1-\sigma_{t} \end{array}).$$ (5)

Here, $c_{i}$ is the i’th constraint, and 1 is a vector of ones.

The Lagrangian L of the above problem is

$$L(\sigma_{t},s,z)=f-\overset{2NM}{\underset{i=1}{\sum }}z_{i}(c_{i}-s_{i})=f-z^{T}(c-s),$$ (6)

where z is a vector of Lagrangian multipliers or dual variables.

The KKT conditions of Eq. (4) with duality gap μ is written as

$$\nabla f-A^{T}z=0,\;Sz-\mu 1=0,\;c-s=0,\;s\geq 0,\;z\geq 0,$$ (7)

where S=diag(s), and

$$A=\nabla c=(\begin{array}{c} \nabla c_{1}\\ \vdots \\ \nabla c_{2NM} \end{array})=(\begin{array}{c} I\\ -I \end{array}).$$ (8)

Here, I is an identity matrix.

To solve the system of the KKT conditions by using Newton’s method, we have the following system of equations:

$$(\begin{array}{ccc} \nabla^{2}L & 0 & -A^{T}\\ 0 & Z & S\\ A & -I & 0 \end{array})(\begin{array}{c} p_{\sigma }\\ p_{s}\\ p_{z} \end{array})=-(\begin{array}{c} \nabla f-A^{T}z\\ Sz-\mu 1\\ c-s \end{array}),$$ (9)

where Z=diag(z) and $\nabla^{2}L$ is the Hessian of the Lagrangian.

### 3.2 Solving the System Efficiently

The matrix in Eq. (9) is of the size 5NM×5NM, which is sparse but large and computationally expensive to solve. We, therefore, develop an efficient way to solve the system by using the problem structure.

First, the system is explicitly written as follows:

$$\left\{ \begin{array}{l} \nabla^{2}Lp_{\sigma }-A^{T}p_{z}=-\nabla f+A^{T}z\\ Zp_{s}+Sp_{z}=-Sz+\mu 1\\ Ap_{\sigma }-p_{s}=-c+s \end{array}.\right.$$ (10)

Substituting the last equation into the second one yields

$$A^{T}S^{-1}Z(Ap_{\sigma }+c-s)+A^{T}p_{z}=-A^{T}z+\mu A^{T}S^{-1}1,$$ (11)

and we add both sides to the first equation to obtain

$$\nabla^{2}Lp_{\sigma }+A^{T}S^{-1}Z(Ap_{\sigma }+c-s)=-A^{T}z+\mu A^{T}S^{-1}1-\nabla f+A^{T}z.$$ (12)

Here, we define $w=s^{-1}⊙z$ and $y=\mu s^{-1}-w⊙c+z$, where ⊙ is the Hadamard (element-wise) product, and $s^{-1}$ is a vector of element-wise reciprocals of s. Then, we have

$$p_{\sigma }={\left(\nabla^{2}L+A^{T}S^{-1}ZA\right)}^{-1}(-\nabla f+A^{T}y),$$ (13)

$$p_{z}=\mu s^{-1}-w⊙p_{s}-z.$$ (14)

By exploiting the structure of matrix A and defining $w={\left(w_{l}^{T},w_{u}^{T}\right)}^{T}$ to split a vector into two parts corresponding to lower and upper bound constraints, we have

$$A^{T}S^{-1}ZA=(\begin{array}{cc} I & -I \end{array})\mathrm{diag}(w)(\begin{array}{c} I\\ -I \end{array})=\mathrm{diag}(w_{l}+w_{u}).$$ (15)

Similarly, we define $y={\left(y_{l}^{T},y_{u}^{T}\right)}^{T}$ to simplify $A^{T}y$ as $A^{T}y=y_{l}-y_{u}$ and then

$$Ap_{\sigma }=(\begin{array}{c} I\\ -I \end{array})p_{\sigma }=(\begin{array}{c} p_{\sigma }\\ -p_{\sigma } \end{array}).$$ (16)

Now, the solution is given by

$$\left\{ \begin{array}{l} p_{\sigma }={\left[\nabla^{2}L+\mathrm{diag}(w_{l}+w_{u})\right]}^{-1}(-\nabla f+y_{l}-y_{u})\\ p_{s}=(\begin{array}{c} p_{\sigma }\\ -p_{\sigma } \end{array})+c-s\\ p_{z}=\mu s^{-1}-w⊙p_{s}-z \end{array},\right.$$ (17)

which involves the inversion of the size NM×NM.

### 3.3 Update Variables

Once $p_{\sigma }$, $p_{s}$, and $p_{z}$ are obtained, we then estimate the step length to update the parameters.^16^ The maximum of the step lengths is given by the following rule:

$$\left\{ \begin{array}{c} \alpha_{s}^{\max}=\max\{\alpha \in [0,1]|s+\alpha p_{s}\geq (1-\tau )s\}\\ \alpha_{z}^{\max}=\max\{\alpha \in [0,1]|z+\alpha p_{z}\geq (1-\tau )z\} \end{array},\right.$$ (18)

with τ∈(0,1) used (for example, τ=0.995). This prevents variables s and z from approaching the lower boundary.

Next, we perform the backtracking line search^30^ to estimate acceptable step lengths $\alpha_{s}$ and $\alpha_{z}$. To this end, we use the following exact merit function ϕ with η∈(0,1):

$$\phi (\sigma_{t},s)=f-\mu \overset{2MN}{\underset{i=1}{\sum }}\log(s_{i})+v\|c(x)-s\|,$$ (19)

and make a sufficient decrease requirement

$$\phi (\sigma_{t}+\alpha_{s}p_{\sigma },s+\alpha_{s}p_{s})\leq \phi (\sigma_{t},s)+\eta \alpha_{s}D_{\phi }(\sigma_{t},s;p_{\sigma },p_{s}),$$ (20)

where $D_{\phi }(\sigma_{t},s;p_{\sigma },p_{s})$ denotes the directional derivative of ϕ in the direction $\left(p_{\sigma },p_{s}\right)$. The step lengths $\alpha_{s}$ and $\alpha_{z}$ are found in the ranges $\alpha_{s}\in (0,\alpha_{s}^{\max}]$ and $\alpha_{z}\in (0,\alpha_{z}^{\max}]$ so that Eq. (20) is satisfied.

Then, the parameters $\sigma_{t}$, s, and z are updated as

$$\left\{ \begin{array}{l} \sigma_{t}\leftarrow \sigma_{t}+\alpha_{s}p_{\sigma }\\ s\leftarrow s+\alpha_{s}p_{s}\\ z\leftarrow z+\alpha_{z}p_{z} \end{array}.\right.$$ (21)

Once the following error function is smaller than a given threshold, the PD interior point method stops

$$E(\sigma_{t},s,z;\mu )=\max\{\|\nabla f-A^{T}z\|,\|Sz-\mu 1\|,\|c-s\|\}.$$ (22)

Algorithm 1 summarizes the PD interior point method developed above. Note that the Hessian $\nabla^{2}L$ can be approximated as $B_{k}$ at each iteration k by the quasi-Newton method, instead of the full Hessian used by Newton’s method. We will compare Newton’s method and the quasi-Newton method in the section of experiments.

**Algorithm 1:** Primal-dual interior point with line search.

**Data:**μ>0, σ∈(0,1), $\varepsilon_{\mathrm{TOL}}>0$, $\varepsilon_{\mu }$, η∈(0,1), k=0.
**Input:** A feasible initial estimates $\sigma_{t}$, s>0, and z>0.
**Input:**$B_{0}=I$ // Only for quasi-Newton
**Result:** Estimates of $\sigma_{t}$
1 **repeat**
2 **repeat** // inner loop
3 Compute the decent direction $p=(p_{\sigma },p_{s},p_{z})$
4 Compute the step lengths $\alpha_{s}$ and $\alpha_{z}$
5 Update $\sigma_{t}$, s, z
6 Update the approximation $B_{k}$ // Only for quasi-Newton
7 Set k←k+1
8 **until**$E(\sigma_{tk},s_{k},z_{k};\mu )\leq \varepsilon_{\mu }$;
9 μ←σμ
10 $\varepsilon_{\mu }\leftarrow \mu$
11 **until**$E(\sigma_{tk},s_{k},z_{k};0)\leq \varepsilon_{\mathrm{TOL}}$;

## 4 Efficient Formulations

The most computationally intensive part of the PD algorithm shown above is the computation of Hessians for Newton’s method and Jacobians for Newton’s and quasi-Newton methods. We propose here efficient formulations of Hessian and Jacobian of the problem, whose computational cost is much smaller than naive formulations used in the previous approaches.

First, we show the naive and old formulations of Hessian and Jacobian, then introduce our new formulations, followed by discussions on computational cost.

### 4.1 Previous Old Formulations for Inverse Problem

Here, we show how the previous approaches^12^^,^^13^^,^^15^ do. We call these the old formulations.

#### 4.1.1 Jacobian: old formulation

Remember that the objective function f to be minimized is

$$f=\overset{N}{\underset{i=1}{\sum }}\overset{N}{\underset{j=1}{\sum }}\left[I_{ij}^{2}-2I_{ij}I_{0}\overset{N_{ij}}{\underset{k=1}{\sum }}H_{ijk}e^{-\sigma_{t}^{T}D_{ijk}}+I_{0}^{2}\overset{N_{ij}}{\underset{k=1}{\sum }}\overset{N_{ij}}{\underset{l=1}{\sum }}H_{ijk}H_{ijl}e^{-\sigma_{t}^{T}(D_{ijk}+D_{ijl})}\right].$$ (23)

The gradient of f is given as follows by taking the derivative of the objective function:

$$\frac{\partial f}{\partial \sigma_{t}}=\overset{N}{\underset{i=1}{\sum }}\overset{N}{\underset{j=1}{\sum }}[2I_{ij}I_{0}\overset{N_{ij}}{\underset{k=1}{\sum }}H_{ijk}e^{-\sigma_{t}^{T}D_{ijk}}D_{ijk}\;-I_{0}^{2}\overset{N_{ij}}{\underset{k=1}{\sum }}\overset{N_{ij}}{\underset{l=1}{\sum }}H_{ijk}H_{ijl}e^{-\sigma_{t}^{T}(D_{ijk}+D_{ijl})}(D_{ijk}+D_{ijl})].$$ (24)

To simplify the equation, the following notations are introduced:

$$E_{ij}=(\begin{array}{c} e^{-\sigma_{t}^{T}D_{ij1}}\\ e^{-\sigma_{t}^{T}D_{ij2}}\\ \vdots \\ e^{-\sigma_{t}^{T}D_{ijN_{ij}}} \end{array}),\;H_{ij}=(\begin{array}{c} H_{ij1}\\ H_{ij2}\\ \vdots \\ H_{ijN_{ij}} \end{array}),\;D_{ij}=(\begin{array}{c} D_{ij1}\\ D_{ij2}\\ \vdots \\ D_{ijN_{ij}} \end{array}),$$ (25)

$$\overset{˜}{D_{ij}}=(\begin{array}{ccc} D_{ij1}+D_{ij1} & \cdots & D_{ij1}+D_{ijN_{ij}}\\ D_{ij2}+D_{ij1} & \cdots & D_{ij2}+D_{ijN_{ij}}\\ \vdots & \ddots & \vdots \\ D_{ijN_{ij}}+D_{ij1} & \cdots & D_{ijN_{ij}}+D_{ijN_{ij}} \end{array}).$$ (26)

Now, f and the gradient are rewritten as follows:

$$f=\overset{N}{\underset{i=1}{\sum }}\overset{N}{\underset{j=1}{\sum }}[I_{ij}^{2}-2I_{ij}I_{0}E_{ij}^{T}H_{ij}+I_{0}^{2}{\left(E_{ij}^{T}H_{ij}\right)}^{2}],$$ (27)

$$\frac{\partial f}{\partial \sigma_{t}}=\overset{N}{\underset{i=1}{\sum }}\overset{N}{\underset{j=1}{\sum }}(2I_{ij}I_{0}\text{sum}[(E_{ij}⊙H_{ij})⊗D_{ij}]-I_{0}^{2}\text{sum}\{[(E_{ij}⊙H_{ij}){\left(E_{ij}⊙H_{ij}\right)}^{T}]⊗\overset{˜}{D_{ij}}\}),$$ (28)

where sum{} stands for the sum over the elements of the container [Eq. (26)] of vectors, and ⊗ denotes the tensor product.

#### 4.1.2 Hessian: old formulation

We define the following notations:

$$\beta_{ijx}=(\begin{array}{c} D_{ij1x}\\ D_{ij2x}\\ \vdots \\ D_{ijkx} \end{array}),$$ (29)

$$\gamma_{ijx}=(\begin{array}{ccc} D_{ij1x}+D_{ij1x} & \cdots & D_{ij1x}+D_{ijkx}\\ D_{ij2x}+D_{ij1x} & \cdots & D_{ij2x}+D_{ijkx}\\ \vdots & \ddots & \vdots \\ D_{ijkx}+D_{ij1x} & \cdots & D_{ijkx}+D_{ijkx} \end{array}),$$ (30)

where $D_{ijkx}$ stands for the x’th element of $D_{ijk}$.

Now, the second-order derivate of f can be represented as follows:

$$\frac{\partial^{2}f}{\partial^{2}\sigma_{t}}=(\begin{array}{cccc} h_{1,1} & h_{1,2} & \cdots & h_{1,NM}\\ h_{2,1} & h_{2,2} & \cdots & h_{2,NM}\\ \vdots & \vdots & \cdots & \vdots \\ h_{NM,1} & h_{NM,2} & \cdots & h_{NM,NM} \end{array}),$$ (31)

where each element is given by

$$h_{xy}=-\overset{N}{\underset{i=1}{\sum }}\overset{N}{\underset{j=1}{\sum }}I_{ij}I_{0}\text{sum}(E_{ij}⊙H_{ij}⊙\beta_{ijx}⊙\beta_{ijy})+\overset{N}{\underset{i=1}{\sum }}\overset{N}{\underset{j=1}{\sum }}I_{0}^{2}\text{sum}[(E_{ij}⊙H_{ij}){\left(E_{ij}⊙H_{ij}\right)}^{T}⊙\gamma_{ijx}⊙\gamma_{ijy}].$$ (32)

### 4.2 Proposed New Efficient Formulation

The problem of the previous old formulations of Jacobian and Hessian shown above is the computation cost increasing as the number $N_{ij}$ of paths increases. As discussed later, the computation cost is $O(N_{ij}^{2})$ on average.

The idea of the proposed formulation is to explore the property of the exponential function and its derivative in the problem. As shown below, the computation cost can be reduced to $O(N_{ij})$ on average.

#### 4.2.1 Jacobian: new formulation

First, we rewrite the cost function as follows:

$$f=\overset{N}{\underset{i=1}{\sum }}\overset{N}{\underset{j=1}{\sum }}r_{ij}^{2},$$ (33)

where $r_{ij}$ is a residual

$$r_{ij}=I_{ij}-I_{0}\overset{N_{ij}}{\underset{k=1}{\sum }}H_{ijk}e^{-\sigma_{t}^{T}D_{ijk}}=I_{ij}-I_{0}E_{ij}^{T}H_{ij}.$$ (34)

Now we use the chain rule of differentiation, and we have

$$\frac{\partial f}{\partial \sigma_{t}}=\overset{N}{\underset{i=1}{\sum }}\overset{N}{\underset{j=1}{\sum }}2r_{ij}\frac{\partial r_{ij}}{\partial \sigma_{t}},$$ (35)

where

$$\frac{\partial r_{ij}}{\partial \sigma_{t}}=I_{0}\overset{N_{ij}}{\underset{k=1}{\sum }}H_{ijk}e^{-\sigma_{t}^{T}D_{ijk}}D_{ijk}=I_{0}D_{ij}(E_{ij}⊙H_{ij}).$$ (36)

Here, we define [Note that this is not the same as the one defined in the previous approaches above, which is a structure used in MATLAB codes to store $D_{ijk}$. Here, $D_{ij}$ is an $(NM)\times N_{ij}$ matrix.]

$$D_{ij}=(\begin{array}{c} D_{ij1},D_{ij2},\ldots ,D_{ijN_{ij}} \end{array}),$$ (37)

which has $N_{ij}$ vectors of dimension NM, and $\left(E_{ij}⊙H_{ij}\right)$ is a coefficient vector. Therefore,

$$\frac{\partial f}{\partial \sigma_{t}}=\overset{N}{\underset{i=1}{\sum }}\overset{N}{\underset{j=1}{\sum }}2I_{0}(I_{ij}-I_{0}E_{ij}^{T}H_{ij})D_{ij}(E_{ij}⊙H_{ij}).$$ (38)

#### 4.2.2 Discussion

Suppose that the expectation of the number of paths is $\bar{N}=E[N_{ij}]$. Then, the computations for the proposed new formulation of Jacobian above are:

• $O(\bar{N})$ multiplications for $E_{ij}⊙H_{ij}$,
• $O(\bar{N})$ additions for $E_{ij}^{T}H_{ij}$,
• $O(\bar{N}NM)$ multiplications and additions for $D_{ij}(E_{ij}⊙H_{ij})$ because there are $O(\bar{N})$ vectors of dimension NM,

for each i and j. In total, it takes $O(\bar{N}N^{3}M)$ operations to compute NM elements of the Jacobian or $O(\bar{N}N^{2})$ operations per element.

Contrary, for each i and j, the previous old formulation Eq. (28) needs:

• $O(\bar{N})$ multiplications for $E_{ij}⊙H_{ij}$,
• $O(\bar{N}NM)$ multiplications for $(E_{ij}⊙H_{ij})⊗D_{ij}$ because there are $O(\bar{N})$ vectors of dimension NM,
• $O(\bar{N}NM)$ additions for $\text{sum}[(E_{ij}⊙H_{ij})⊗D_{ij}]$,

for the first term, and

• $O({\bar{N}}^{2})$ multiplications for $(E_{ij}⊙H_{ij}){\left(E_{ij}⊙H_{ij}\right)}^{T}$,
• $O({\overset{¯}{N}}^{2}NM)$ additions for computing $\overset{˜}{D_{ij}}$ because there are $O({\bar{N}}^{2})$ vectors of dimension NM,
• $O({\bar{N}}^{2}NM)$ multiplications for $[(E_{ij}⊙H_{ij}){\left(E_{ij}⊙H_{ij}\right)}^{T}]⊗\overset{˜}{D_{ij}}$,
• $O({\overset{¯}{N}}^{2}NM)$ additions for $\text{sum}\{[(E_{ij}⊙H_{ij}){\left(E_{ij}⊙H_{ij}\right)}^{T}]⊗\overset{˜}{D_{ij}}\}$,

for the second term. In total, it takes $O({\overset{¯}{N}}^{2}N^{3}M)$ operations to compute NM elements of the Jacobian, or $O({\overset{¯}{N}}^{2}N^{2})$ operations per element. The difference is mainly caused by the second term of Eq. (28).

In summary, the proposed new formulation has the cost of $O(\bar{N}N^{2})$ operations per element, whereas the previous old formulation has the cost of $O({\overset{¯}{N}}^{2}N^{2})$ operations per element. Table 1 summarizes the discussion above.

**Table 1:** Comparison of the new and old formulations for computing the Jacobian.

Terms	New	Old
$E_{ij}⊙H_{ij}$	$O(\overset{¯}{N})$	$O(\bar{N})$
$E_{ij}^{T}H_{ij}$	$O(\bar{N})$	
$D_{ij}(E_{ij}⊙H_{ij})$	$O(\overset{¯}{N}NM)$	
$(E_{ij}⊙H_{ij})⊙D_{ij}$		$O(\overset{¯}{N}NM)$
$\text{sum}[(E_{ij}⊙H_{ij})⊙D_{ij}]$		$O(\bar{N}NM)$
$(E_{ij}⊙H_{ij}){\left(E_{ij}⊙H_{ij}\right)}^{T}$		$O({\bar{N}}^{2})$
$\overset{˜}{D_{ij}}$		$O({\bar{N}}^{2}NM)$
$[(E_{ij}⊙H_{ij}){\left(E_{ij}⊙H_{ij}\right)}^{T}]⊙\overset{˜}{D_{ij}}$		$O({\bar{N}}^{2}NM)$
$\text{sum}\{[(E_{ij}⊙H_{ij}){\left(E_{ij}⊙H_{ij}\right)}^{T}]⊙\overset{˜}{D_{ij}}\}$		$O({\bar{N}}^{2}NM)$
Per element	$O(\bar{N}N^{2})$	$O({\overset{¯}{N}}^{2}N^{2})$

#### 4.2.3 Hessian: new formulation

In the same manner, we can derive the Hessian as follows. From the Jacobian

$$\frac{\partial f}{\partial \sigma_{t}}=\overset{N}{\underset{i=1}{\sum }}\overset{N}{\underset{j=1}{\sum }}2r_{ij}\frac{\partial r_{ij}}{\partial \sigma_{t}},$$ (39)

we obtain the Hessian by using the chain rule of differentiation

$$\frac{\partial^{2}f}{\partial \sigma_{t}^{2}}=\overset{N}{\underset{i=1}{\sum }}\overset{N}{\underset{j=1}{\sum }}2\frac{\partial r_{ij}}{\partial \sigma_{t}}{\frac{\partial r_{ij}}{\partial \sigma_{t}}}^{T}+2r_{ij}\frac{\partial^{2}r_{ij}}{\partial \sigma_{t}^{2}},$$ (40)

where

$$\frac{\partial^{2}r_{ij}}{\partial \sigma_{t}^{2}}=-I_{0}\overset{N_{ij}}{\underset{k=1}{\sum }}H_{ijk}e^{-\sigma_{t}^{T}D_{ijk}}D_{ijk}D_{ijk}^{T},$$ (41)

$$=-I_{0}D_{ij}\mathrm{diag}(E_{ij}⊙H_{ij})D_{ij}^{T}.$$ (42)

Now, the Hessian can be written as follows:

$$\frac{\partial^{2}f}{\partial \sigma_{t}^{2}}=\overset{N}{\underset{i=1}{\sum }}\overset{N}{\underset{j=1}{\sum }}2I_{0}^{2}D_{ij}(E_{ij}⊙H_{ij}){\left[D_{ij}(E_{ij}⊙H_{ij})\right]}^{T}\;-2I_{0}(I_{ij}-I_{0}E_{ij}^{T}H_{ij})D_{ij}\mathrm{diag}(E_{ij}⊙H_{ij})D_{ij}^{T}.$$ (43)

Note that $D_{ij}(E_{ij}⊙H_{ij}){\left[D_{ij}(E_{ij}⊙H_{ij})\right]}^{T}$ should not be expanded as $D_{ij}(E_{ij}⊙H_{ij}){\left(E_{ij}⊙H_{ij}\right)}^{T}D_{ij}^{T}$ because it involves a large matrix $(E_{ij}⊙H_{ij}){\left(E_{ij}⊙H_{ij}\right)}^{T}$, which is computationally intensive to compute.

#### 4.2.4 Discussion

By reusing $E_{ij}⊙H_{ij}$ and $D_{ij}(E_{ij}⊙H_{ij})$ computed for the Jacobian, the new formulation of Hessian needs:

• $O(N^{2}M^{2})$ multiplications for $D_{ij}(E_{ij}⊙H_{ij}){\left[D_{ij}(E_{ij}⊙H_{ij})\right]}^{T}$,
• $O(\overset{¯}{N}NM)$ multiplications for $D_{ij}\mathrm{diag}(E_{ij}⊙H_{ij})$,
• $O(\overset{¯}{N}N^{2}M^{2})$ multiplications for $D_{ij}\mathrm{diag}(E_{ij}⊙H_{ij})D_{ij}^{T}$,

for each i and j. In total, it takes $O(\overset{¯}{N}N^{4}M^{2})$ operations to compute $N^{2}M^{2}$ elements of the Hessian or $O(\overset{¯}{N}N^{2})$ operations per element.

Contrarily, for each element, the previous formulation [Eq. (32)] needs:

• $O({\bar{N}}^{2})$ multiplications for $\gamma_{ijx}$,
• $O(\bar{N})$ multiplications and the sum for $\left(E_{ij}⊙H_{ij}⊙\beta_{ijx}⊙\beta_{ijy}\right)$,
• $O({\bar{N}}^{2})$ multiplications for $(E_{ij}⊙H_{ij}){\left(E_{ij}⊙H_{ij}\right)}^{T}$,
• $O({\bar{N}}^{2})$ multiplications and the sum for $(E_{ij}⊙H_{ij}){\left(E_{ij}⊙H_{ij}\right)}^{T}⊙\gamma_{ijx}⊙\gamma_{ijy}$,
• $O({\bar{N}}^{2}N^{2})$ additions for the sums of i and j.

In total, it takes $O({\bar{N}}^{2}N^{2})$ operations to compute a single element of the Hessian.

In summary, the proposed new formulation has the cost of $O(\bar{N}N^{2})$ operations per element, whereas the previous old formulation has the cost of $O(N^{2}{\bar{N}}^{2})$ operations per element. Table 2 summarizes the discussion above.

**Table 2:** Comparison of the new and old formulations for computing the Hessian.

Terms	New	Old
$D_{ij}(E_{ij}⊙H_{ij}){\left[D_{ij}(E_{ij}⊙H_{ij})\right]}^{T}$	$O(N^{2}M^{2})$	
$D_{ij}\mathrm{diag}(E_{ij}⊙H_{ij})$	$O(\overset{¯}{N}NM)$	
$D_{ij}\mathrm{diag}(E_{ij}⊙H_{ij})D_{ij}^{T}$	$O(\overset{¯}{N}N^{2}M^{2})$	
$\left(E_{ij}⊙H_{ij}⊙\beta_{ijx}⊙\beta_{ijy}\right)$		$O(\bar{N})$
$(E_{ij}⊙H_{ij}){\left(E_{ij}⊙H_{ij}\right)}^{T}$		$O({\overset{¯}{N}}^{2})$
$(E_{ij}⊙H_{ij}){\left(E_{ij}⊙H_{ij}\right)}^{T}⊙\gamma_{ijx}⊙\gamma_{ijy}$		$O({\bar{N}}^{2})$
Sums of i, j		$O(N^{2}{\bar{N}}^{2})$
Per element	$O(\bar{N}N^{2})$	$O(N^{2}{\overset{¯}{N}}^{2})$

## 5 Numerical Simulation

In this section, we report the results obtained by simulations using the proposed method by comparing PD and LB interior point methods, as well as old and new formulations of Jacobian and Hessian.

Since the mathematical model we used to describe the light transport in the forward problem is exactly the same as the model in the previous work,^15^ we use the same setup as follows. For the 2-D layered medium, the grid size was set to N=M=24 with square voxels of size 1 (mm), i.e., the medium is 24  (mm)×24  (mm). The values of the extinction coefficients are set between 1.05 and 1.55 (${\mathrm{mm}}^{-1}$), and the lower and upper bounds ($\sigma_{tl}$ and $\sigma_{tu}$) are set to be 1.0 and 2.0 (${\mathrm{mm}}^{-1}$), respectively. Values of the initial guess are 1.001 for all elements of $\sigma_{t0}$, as well as $s_{0}$ and $z_{0}$. Parameters used in Algorithm 1 are set as σ=0.5, η=0.01, $\varepsilon_{\mu }=1$, and $\varepsilon_{\mathrm{TOL}}=0.02$.

### 5.1 Estimation Quality

**Fig. 3:**
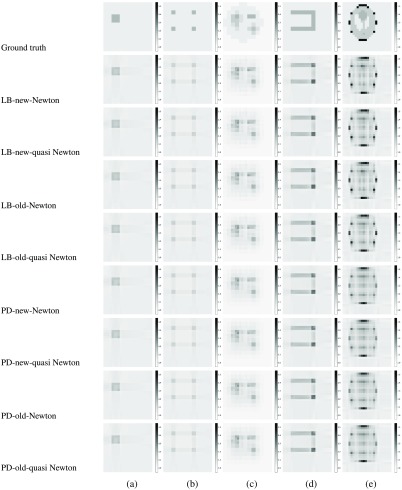
Numerical simulation results for five media (a)–(e) in a grid of 24×24. Darker shades of gray represent larger values of extinction coefficients (more light is absorbed at the voxel). The bars on the side show extinction coefficient values in gray scale. The first row shows ground truth for five different types of media used for the simulation. The following rows show the estimated results for different combinations of LB or PD methods, old or new formulas, and Newton’s or quasi-Newton methods.

The ground truth and the estimated results are shown in Fig. 3. The matrix plots in the top row of this figure represent five different media (a)–(e) used for the simulation, which were also used in the previous work.^15^ Note that the medium e is the Shepp–Logan phantom.^31^ Each voxel is shaded in gray according to the values of the extinction coefficients.

**Fig. 4:**
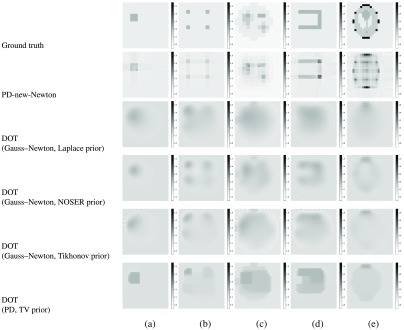
Numerical simulation results for five media (a)–(e) in a grid of 24×24. Darker shades of gray represent larger values of extinction coefficients (more light is absorbed at the voxel). The bars on the side show extinction coefficient values in gray scale. The first row shows ground truth for five different types of media used for the simulation. The following rows show the estimated results for different combinations of LB or PD methods, old or new formulas, and Newton’s or quasi-Newton methods. Results of the previous work^15^ and DOT are shown as baselines in the last three rows.

The following rows show the estimated results of different combinations of LB or PD methods, old or new formulas, and Newton’s or quasi-Newton methods. The proposed method is PD-new-Newton/quasi-Newton; that is, the PD method with Newton’s or quasi-Newton method by using the proposed new formulation. The row LB-old-quasi-Newton corresponds to the previous work^15^ that uses the LB method with the quasi-Newton method by using the old formulation, and the row LB-old-Newton corresponds to another prior work.^12^

As we can see, the results of different combinations almost look the same for each of the five media. This observation is also validated by the root-mean-square error (RMSE) shown in Table 3. The RMSE values of all combinations are more or less the same, while some variations appear due to the different update rules between Newton’s and quasi-Newton methods, and different stopping conditions between LB and PD methods.

**Table 3:** RMSEs and computation time for the numerical simulations for five different types of media (a)–(e) with grid size of 24×24, for different combinations of LB or PD methods, old or new formulas, and Newton’s or quasi-Newton methods. Each computation time shows the mean and standard deviation of 10 trials, except the combinations of “old-Newton.” Note that RMSE values are exactly the same for 10 trials. Results of DOT methods are shown for comparison.

		a	b	c	d	e
RMSE	LB-new-Newton	0.008422	0.012643	0.014594	0.021246	0.052511
	LB-new-quasi-Newton	0.008646	0.012478	0.014444	0.020375	0.049811
	LB-old-Newton	0.008422	0.012643	0.014594	0.021246	0.052420
	LB-old-quasi-Newton	0.008646	0.012478	0.014444	0.020375	0.049818
	PD-new-Newton	0.009776	0.013190	0.014490	0.021251	0.055912
	PD-new-quasi-Newton	0.009754	0.013184	0.014502	0.021201	0.056085
	PD-old-Newton	0.009776	0.013190	0.014490	0.021251	0.055912
	PD-old-quasi-Newton	0.009754	0.013184	0.014502	0.021201	0.056084
	DOT (GN, Laplace prior)	0.059339	0.062984	0.078100	0.065001	0.087094
	DOT (GN, NOSER prior)	0.052053	0.057515	0.075478	0.059156	0.086397
	DOT (GN, Tikhonov prior)	0.054729	0.056196	0.073146	0.059284	0.087659
	DOT (PD, TV prior)	0.055047	0.059219	0.081811	0.070263	0.086107
Computation time (s)	LB-new-Newton	60.00±4.60	57.63±1.41	61.90±2.88	62.32±1.22	93.10±2.46
	LB-new-quasi-Newton	18.64±0.90	17.22±1.03	25.32±1.21	20.73±1.10	32.57±0.58
	LB-old-Newton	126100	12848	13383	14037	21577
	LB-old-quasi-Newton	44.86±1.33	42.58±1.19	63.75±1.76	54.05±2.04	91.17±1.48
	PD-new-Newton	18.73±2.18	16.52±0.67	17.40±0.93	18.28±1.30	23.14±1.32
	PD-new-quasi-Newton	14.44±0.78	13.07±0.61	40.25±1.01	30.77±0.86	48.26±1.59
	PD-old-Newton	5673	5418	5824	5663	7547
	PD-old-quasi-Newton	75.67±1.34	67.18±1.38	203.42±3.42	155.69±2.67	248.05±4.86
	DOT (GN, Laplace prior)	0.34±0.04	0.41±0.04	0.40±0.03	0.40±0.04	0.40±0.03
	DOT (GN, NOSER prior)	0.52±0.04	0.52±0.05	0.53±0.05	0.50±0.01	0.52±0.03
	DOT (GN, Tikhonov prior)	0.29±0.01	0.29±0.02	0.30±0.03	0.29±0.01	0.29±0.02
	DOT (PD, TV prior)	2.67±0.07	2.68±0.06	2.67±0.07	2.64±0.05	2.66±0.07

### 5.2 Estimation Time

The main goal of this paper is to develop an efficient way to solve the inverse problem. Table 3 shows the computation cost of different combinations. All experiments were performed on a Linux workstation (two Intel Xeon E5-2630 2.4 GHz CPUs, total 16 physical cores, with 256 GB memory). We implemented the method in MATLAB R2017a and did not explicitly use the parallel computation toolbox of MATLAB except the Hessian computation of LB/PD-old-Newton due to its slow computation. Parallel matrix multiplications are, however, automatically performed on MATLAB. Table 3 shows the computation time for different computations in seconds. We report the average and standard deviation of 10 trials, except the cases of LB/PD-old-Newton which show the processing time of a single trial.

For any combination, our proposed new formulation is much faster than the old formulation. The uses of Newton’s method greatly benefit from the efficient Hessian computation and the computation time reduces more than a factor of 100. However, the new formulation does not help to reduce the computation cost of quasi-Newton methods and the reduction is a factor of just 2 or 3. This is due to the fact that the quasi-Newton method needs gradient vectors, and its computation is of the order NM (the number of voxels) and it is not so large in terms of the total computation cost. In contrast, Hessian computation in the Newton’s method is of the size NM×NM, which is quite large compared to the gradient. Our new formulation is, therefore, better when the Newton method is used.

With the quasi-Newton method, the PD approach seems to be comparable to the LB method. By comparing rows LB/PD-new-quasi-Newton, LB is faster than PD for denser media (c), (d), and (e). This might be caused by the different ways of approximations by the quasi-Newton method. For the LB method, the gradient is modified by the approximated Hessian. For the PD method, however, the approximated Hessian is used in the matrix to be solved, resulting in an update rule of $p_{\sigma }$ regularized by diagonal elements of w in Eq. (17). Except the simplest medium (a), the fast combination is PD-new-Newton, which is proposed in this paper. This is due to the fast convergence of Newton’s method compared to the quasi-Newton method, and also the fact that the PD method needs fewer iterations than the LB method. The qualities of results are almost the same as discussed above, then PD-new-Newton is the best when the working memory is enough for storing the Hessian. Otherwise, LB/PD-new-quasi-Newton is better to be used.

### 5.3 Comparison

We compare our methods to a standard DOT implemented in the Electrical Impedance Tomography and Diffuse Optical Tomography Reconstruction Software (EIDORS).^32^^,^^33^ in the same setting as the previous work:^15^ N=M=24 medium of size 24  (mm)×24  (mm) with the five media (a)–(e). For solving DOT by EIDORS, we used 24×24×24=1152 triangle elements. For boundary conditions, we placed 48 light sources and detectors at the same intervals around the medium. We used some different solvers and priors; Gauss–Newton method^34^ with Laplace, NOSER,^35^ and Tikhonov priors, and PD method with total variation prior.

Due to the diffusion approximation of DOT, the results in Fig. 4 for DOT with the Gauss–Newton method are blurred, and those for DOT with PD have a tendency of overestimating the high-coefficient value areas. In contrast, the results of PD-new-Newton (and other combinations in Fig. 3) are clearer and sharper for all combinations. This observation is also validated by the RMSE shown in Table 3. The RMSE values of PD-new-Newton are smaller than the values of DOT for all five media.

The obvious drawback of PD-new-Newton is its high computation cost. It is slower by a factor of 10 compared to DOT with the PD method and a factor of 100 to DOT with the Gauss–Newton method. A large part of the computation cost comes from the computation of Hessian and Jacobian, which depends on the number of paths $N_{ij}$. Further reduction of computation cost is left for our future work.

## 6 Conclusion

In this paper, we have proposed a PD approach to optical tomography with a discretized path integral and also efficient formulation for computing Hessian and Jacobian. Numerical simulation examples with 2-D layered media are shown to demonstrate that the proposed method, called PD-new-Newton in the experiments, performed faster than the previous work (LB-old-quasi-Newton) while the estimated extinction coefficients of both methods were comparable. Compared to DOT, the proposed method worked slower but produced better estimation results in terms of RMSE.
